# Epigenetic heredity of human height

**DOI:** 10.14814/phy2.12047

**Published:** 2014-06-24

**Authors:** Pasquale Simeone, Saverio Alberti

**Affiliations:** 1 Unit of Cancer Pathology, Department of Neuroscience and Imaging and CeSI, University “G. d'Annunzio” Foundation, Chieti Scalo, Italy

**Keywords:** Bioinformatics, DNA methylation, epigenetic regulation, genome‐wide association studies, meta‐analysis

## Abstract

Genome‐wide SNP analyses have identified genomic variants associated with adult human height. However, these only explain a fraction of human height variation, suggesting that significant information might have been systematically missed by SNP sequencing analysis. A candidate for such non‐SNP‐linked information is DNA methylation. Regulation by DNA methylation requires the presence of CpG islands in the promoter region of candidate genes. Seventy two of 87 (82.8%), height‐associated genes were indeed found to contain CpG islands upstream of the transcription start site (USC CpG island searcher; validation: UCSC Genome Browser), which were shown to correlate with gene regulation. Consistent with this, DNA hypermethylation modules were detected in 42 height‐associated genes, versus 1.5% of control genes (P = 8.0199e^−17^), as were dynamic methylation changes and gene imprinting. Epigenetic heredity thus appears to be a determinant of adult human height. Major findings in mouse models and in human genetic diseases support this model. Modulation of DNA methylation are candidate to mediate environmental influence on epigenetic traits. This may help to explain progressive height changes over multiple generations, through trans‐generational heredity of progressive DNA methylation patterns.

## Abstract (precis)

Epigenetic heredity appears to be a determinant of adult human height. Major findings in mouse models and in human genetic diseases support this model. Modulation of DNA methylation is candidate to mediate environmental influence on epigenetic traits.

## Introduction

Genomic loci linked to human height have been identified by genome‐wide SNP‐association analyses (GWAS) (Gudbjartsson et al. 2008; Lettre et al. 2008; Weedon et al. 2008). The findings from different studies overlap substantially (Table 1, Table S1), strongly supporting identification of a significant fraction of height‐determining genes. Cho et al. (2009) performed a corresponding GWAS in a cohort of Korean descent. Notably, fifteen of the genes identified (ACAN, BCAS3 also known as TBX2, EFEMP1, HHIP, HMGA1, HMGA2, LCORL, NCAPG, PLAGL1, PTCH1, SOCS2, SPAG1, UQCC also known as GDF5, ZBTB38, ZNF678) were also identified in the previous studies (Weedon and Frayling 2008; Cho et al. 2009). Correspondingly, the Caucasian height‐associated genes were confirmed as such in cohorts of Japanese descent (Okada et al. 2010). These findings showed that the same genes are largely associated with height in populations of both Caucasian and non‐Caucasian ancestry, and that inherited height‐controlling mechanisms are conserved. Genes close to the SNP most strongly associated with body size were shown to encode extracellular matrix components, proteases, cell cycle controllers, transcription factors and signaling molecules [(Gudbjartsson et al. 2008; Lettre et al. 2008; Weedon et al. 2008) and manuscript in prep.].

**Table 1.** CpG islands in human height‐associated gene promoters.

*Genes*	RefSeq number	Chromosome	TSS (nt)	Upstream TSS^1^							
				CpG islands	% G+C	obsCpG/expCpG	Length (nt)	Genome sequence assembly		nt upstream TSS	Notes^2^
								5' (nt)	3' (nt)		
*ACAN* (Weedon et al. 2008; Cho et al. 2009)	NM_013227	15	87147678	1	65.7	0.72	1485	87146790	87148274	888	‡
*ADAMTSL3* (Gudbjartsson et al. 2008; Lettre et al. 2008; Weedon et al. 2008)	NM_207517	15	82113842	1	68,7	0,82	1974	82113076	82115050	766	‡
*ADAMTS17* (Gudbjartsson et al. 2008)	NM_139057	15	98699706	2	60,7	0,65	888	9908373	9907485	8667	*
					69,1	0,89	2026	98700323	98698297	617	‡
*AGPAT6* (Lettre et al. 2008)	NM_178819	8	41554876	None							
*ANAPC13* (Weedon et al. 2008)	NM_155391	3	135687519	1	59,2	0,788	1548	135688471	135686923	952	‡
*ANKS1* (Gudbjartsson et al. 2008)	NM_015245	6	34965016	2	55	0,80	660	349633552	349634012	1664	
					61,7	0,78	1497	34964519	34966016	497	‡
*ATAD5* (Gudbjartsson et al. 2008)	NM_024857	17	26183149	2	62,6	0,78	1368	26175115	26176483	8031	*
					56,3	0,812	980	26182826	26183806	320	‡
*ATXN3* (Gudbjartsson et al. 2008)	NM_030660	14	91642718	1	60,4	0,744	1226	91643223	91641997	516	‡
*BCAS3* (Gudbjartsson et al. 2008; Cho et al. 2009)	NM_001099432	17	56109954	2	55	0,70	820	56104151	56104971	5803	*
					59,5	0,65	1058	56109366	56110424	588	‡
*BMP2* (Gudbjartsson et al. 2008)	NM_001200	20	6696745	1	66,7	0,95	1988	6695759	6696745	986	‡
*BMP6* (Gudbjartsson et al. 2008)	NM_001718	6	7672010	1	66,8	0,87	2687	7670904	7673591	1106	‡
*CABLES1* (Gudbjartsson et al. 2008)	NM_001107404	18	18969725	1	67,4	0,95	2620	18968684	18971304	1041	‡
*CDK6* (Gudbjartsson et al. 2008; Lettre et al. 2008; Weedon et al. 2008)	NM_001259	7	92301148	2	61,2	0,748	2768	92304527	92301759	3379	
					62,1	0,819	1951	92301759	92299808	611	‡
*CENTA2* (Gudbjartsson et al. 2008)	NM_018404	17	26272880	2	55,1	0,69	532	26263024	26263556	9856	*
					65,9	0,74	1687	26272611	26274298	269	‡
*CHCHD7* (Gudbjartsson et al. 2008; Lettre et al. 2008)	NM_024300	8	57286869	1	63,7	0,893	1970	57285833	57287803	1036	‡
*COIL* (Gudbjartsson et al. 2008)	NM_004645	17	52393410	1	56,3	0,772	1404	52393685	52392281	275	‡
*CPSF2* (Gudbjartsson et al. 2008)	NM_175808	13	91658090	1	58	0,8	2129	91656710	91658839	1372	‡
*CRLF3* (Gudbjartsson et al. 2008)	NM_015986	17	26175904	2	55,9	0,817	1084	26183907	26182823	8003	*
					62,6	0,775	1368	26176480	26175112	576	‡
*DCC* (Lettre et al. 2008)	NM_005215	18	48120569	1	53,9	0,66	1324	4812170	4813494	399	‡
*DEF6* (Gudbjartsson et al. 2008)	NM_022047	6	35373573	None							
*DGKE* (Gudbjartsson et al. 2008)	NM_003647	17	52266552	1	66,7	0,88	2554	52265265	52267819	1287	‡
*DLEU7* (Weedon et al. 2008)	NM_198989	13	50315886	1	66,4	0,657	1217	50316345	50315128	459	‡
*DNM3* (Gudbjartsson et al. 2008)	NM_015569	1	170077261	1	66,1	0,73	1498	170076559	170078057	702	‡
*DNMT3A* (Gudbjartsson et al. 2008)	NM_175629	2	25342590	1	79,2	0,804	2612	25340227	25342838	248	‡
*DOT1L* (Lettre et al. 2008)	NM_032482	19	2115148	4	55	0,68	505	2108150	21081655	6998	*
					55	0,75	498	2110214	2110712	4934	*
					56,1	0,65	586	2113068	2113654	2080	
					65,3	0,86	2891	2114184	2117075	964	‡
*DYM* (Weedon et al. 2008)	NM_017653	18	45241077	1	60,2	0,808	1397	45241580	45240183	503	‡
*EFEMP1* (Gudbjartsson et al. 2008; Weedon et al. 2008; Cho et al. 2009)	NM_004105	2	56003860	1	58,7	0,683	1350	56004981	56003631	545	‡
*E4F1* (Lettre et al. 2008)	NM_004424	16	2213568	2	64,3	0,92	2647	2204610	2207527	8958	*
					65,1	0,79	1517	2212727	2214244	841	‡
*FBLN5* (Gudbjartsson et al. 2008; Lettre et al. 2008)	NM_006329	14	91483799	1	57,2	0,77	1357	91484395	91483038	607	‡
*FBP2* (Cho et al. 2009)	NM_003837.2	9	97321226	None							
*FUBP3* (Lettre et al. 2008)	NM_003934	9	132444781	1	65,2	0,77	1356	132444169	132445525	612	‡
*GATAD1* (Gudbjartsson et al. 2008)	NM_021167	7	91914701	1	60,8	0,80	1442	91914227	91915669	474	‡
*GLT25D2* (Gudbjartsson et al. 2008)	NM_015101	1	182273486	1	65,1	0,771	1841	182273758	182271917	272	‡
*GNA12* (Gudbjartsson et al. 2008)	NM_007353	7	2850485	1	67,2	0,888	1553	2851125	2849572	640	‡
*GPR126* (Gudbjartsson et al. 2008; Lettre et al. 2008)	NM_001032395	6	142664749	1	61,6	0,73	1488	142663983	142665471	776	‡
*GRB10* (Lettre et al. 2008)	NM_001001549	7	50767544	1	67,5	0,965	1964	50829436	50827472	784	‡
*HHIP* (Gudbjartsson et al. 2008; Lettre et al. 2008; Weedon et al. 2008; Cho et al. 2009)	NM_022475	4	145786623	1	62,2	0,70	1869	145785471	145787340	1152	‡
*HIST1H1D* (Lettre et al. 2008)	NM_005320	6	26343195	1	55	0,667	500	26349982	26349482	6787	*
*HMGA1* (Gudbjartsson et al. 2008; Cho et al. 2009)	NM_145899	6	34312628	1	67,6	0,79	4212	34310234	34314446	2394	‡
*HMGA2* (Gudbjartsson et al. 2008; Lettre et al. 2008; Weedon et al. 2008; Cho et al. 2009)	NM_003483	12	64504507	1	64,4	0,78	3133	64503504	64506637	1003	‡
*IHH* (Weedon et al. 2008)	NM_002191	2	219633433	1	70,2	0,765	1908	219634655	219632747	1222	‡
*LBH* (Gudbjartsson et al. 2008)	NM_030915	6	30307901	1	65,8	0,80	1965	30306777	30308742	1124	‡
*LCORL* (Gudbjartsson et al. 2008; Weedon et al. 2008; Cho et al. 2009)	NM_153686	4	17632483	1	63,8	0,887	2339	17633555	17631216	1078	‡
*LIN28B* (Lettre et al. 2008)	NM_001004317	6	105511616	1	55,1	0,89	490	105507480	105507970	3986	
*LTBP1* (Cho et al. 2009)	NM_206943.2	2	452207	1	65,2	0,909	1292	11650045	11651337	10000	*
*LYAR (Lettre* et al. 2008)	NM_017816	4	4342744	1	61,9	0,69	1379	4343686	4342307	942	‡
*LYN* (Gudbjartsson et al. 2008)	NM_001111097	8	56954940	1	64,9	0,81	1813	56954403	56954926	523	‡
*MOS* (Gudbjartsson et al. 2008)	NM_022746	8	57189095	2	55	0,945	980	57193889	57192909	3994	
					59,9	0,747	1196	57189249	57188053	154	‡
*MTMR11* (Gudbjartsson et al. 2008)	NM_181873	1	148174867	None							
*NACA2* (Gudbjartsson et al. 2008)	NM_199290	17	57023345	None							
*NCAPG* (Gudbjartsson et al. 2008; Cho et al. 2009)	NM_022346	4	17421623	1	59,3	0,89	1574	17420886	17422460	737	‡
*NOG* (Gudbjartsson et al. 2008)	NM_005450	17	52026059	1	64,8	0,79	2982	52024564	52027546	1710	‡
*NKX2‐1/TTF1* (Lettre et al. 2008)	NM_003317	14	36055353	3	60,3	0,67	1661	36064854	36063193	5687	*
					58,5	0,65	1190	36062574	36061384	3407	
					59,4	0,729	2163	36061089	36058926	1922	‡
*PAPPA* (Lettre et al. 2008)	NM_002581	9	117955892	1	64,5	0,85	1373	117955869	117957242	23	‡
*PENK* (Gudbjartsson et al. 2008)	NM_006211	8	57521143	2	57,8	0,65	536	57523392	57522856	2249	‡
					62,2	0,801	2361	57522705	57520344	1562	‡
*PEX1* (Gudbjartsson et al. 2008)	NM_000466	7	91995781	1	59,1	0,928	1356	91996364	91995008	583	‡
*PLAGL1* (Gudbjartsson et al. 2008; Cho et al. 2009)	NM_002655	8	57286413	1	63,7	0,893	1970	57287800	57285830	1408	‡
*PNPT1* (Gudbjartsson et al. 2008)	NM_033109	2	55774515	1	56,1	0,788	909	55774741	55773832	278	‡
*PRKG2* (Lettre et al. 2008)	NM_006259	4	82345239	1	57	0,65	574	82354957	82353513	9718	*
*PTCH1* (Weedon et al. 2008; Cho et al. 2009)	NM_001083605	9	97319068	1	63,6	0,851	2411	97319965	97317554	897	‡
*PXMP3* (Gudbjartsson et al. 2008)	NM_001079867	8	78075079	1	58,6	0,79	1303	78075931	78074628	852	‡
*RAB40C* (Lettre et al. 2008)	NM_021168	16	580180	1	69,7	0,91	2715	578292	581007	1885	‡
*RBBP8* (Gudbjartsson et al. 2008)	NM_203291	18	18767837	1	58,7	0,90	1476	18766793	18768269	500	‡
*RDHE2* (Gudbjartsson et al. 2008; Lettre et al. 2008)	NM_138969	8	57395795	None							
*RNF135* (Gudbjartsson et al. 2008)	NM_197939	17	26322082	1	59,2	0,77	1234	26321702	26322936	380	‡
*RPS20* (Gudbjartsson et al. 2008)	NM_001023	8	57149623	1	55,1	0,951	1047	57150084	57149037	461	‡
*SCMH1* (Weedon et al. 2008)	NM_012236	1	41480375	1	68,7	0,798	1999	41481234	41479235	859	‡
*SCUBE3* (Gudbjartsson et al. 2008)	NM_152753	6	35290168	1	63,1	0,77	2003	35288777	35290780	1391	‡
*SF3B4* (Gudbjartsson et al. 2008)	NM_005850	1	148166326	1	56,1	0,651	879	148166783	148165904	457	‡
*SH3GL3* (Lettre et al. 2008)	NM_003027	15	81907287	1	62,6	0,73	1773	81906488	81908261	799	‡
*SOCS2* (Gudbjartsson et al. 2008; Weedon et al. 2008; Cho et al. 2009)	NM_003877	12	92487729	1	66,4	0,82	2790	92487649	92490439	80	‡
*SPAG1* (Weedon et al. 2008; Cho et al. 2009)	NM_003114	8	101239832	1	58,1	0,77	1239	101238989	101240228	450	‡
*SV2A* (Gudbjartsson et al. 2008)	NM_014849	1	148156054	None							
*TBX2* (Gudbjartsson et al. 2008)	NM_005994	17	56832039	1	67,3	0,81	5974	56827581	56833555	4458	‡
*TBX4* (Gudbjartsson et al. 2008)	NM_001003006	17	56888634	3	67,1	0,74	1700	56883545	56885245	5044	*
					62,3	0,81	1298	56886293	56887591	2296	
					64,6	0,72	1516	56888304	56889820	285	‡
*TCP11* (Gudbjartsson et al. 2008)	NM_018679	6	35217165	1	67,1	0,718	1134	35217617	35216483	452	‡
*TGS1* (Gudbjartsson et al. 2008)	NM_024831	8	56848345	1	59,5	0,8	1400	56847635	56849035	710	‡
*TMED3* (Lettre et al. 2008)	NM_007364	15	77390546	1	66,8	0,71	1133	77389985	77391118	561	‡
*TRIM25* (Gudbjartsson et al. 2008)	NM_005082	17	52346408	1	63,6	0,751	1460	52347061	52345601	653	‡
*TRIP11* (Gudbjartsson et al. 2008; Lettre et al. 2008)	NM_004239	14	91576570	1	56,4	0,768	1310	91576732	91575422	593	‡
*UQCC/GDF5* (Gudbjartsson et al. 2008; Lettre et al. 2008; Weedon et al. 2008; Cho et al. 2009)	NM_018244	20	33463247	1	56,3	0,651	774	33463669	33462895	422	‡
*WDR60* (Lettre et al. 2008)	NM_01851	7	158342030	1	60,7	0,82	1440	158341361	158342801	669	‡
*ZBTB38* (Gudbjartsson et al. 2008; Lettre et al. 2008; Weedon et al. 2008; Cho et al. 2009)	NM_001080412	3	142525745	None							
*ZFHX4* (Gudbjartsson et al. 2008)	NM_024721	8	77756070	3	55	0,78	540	77748585	77749125	7485	*
					56,6	0,72	1064	77752435	77743499	3635	
					55	0,85	545	77755726	77746271	344	‡
*ZNF76* (Gudbjartsson et al. 2008)	NM_003427	6	35335488	1	64,5	0,78	1490	35334502	35335992	986	‡
*ZNF462* (Gudbjartsson et al. 2008)	NM_021224	9	108665199	2	55,4	0,72	1475	108661904	108663379	3295	
					61,9	0,65	507	108664296	108664803	903	
*ZNF678* (Weedon et al. 2008; Cho et al. 2009)	NM_178549	1	225817867	1	66,4	0,66	1300	225814864	108666164	3003	

^1^ TSS: trascription start site. CpG islands were searched for in the region upstream the TSS, within an upper limit of ‐ 10,000 bp.

^2^ ^‡^CpG islands that overlap the TSS;*CpG islands in the region −10,000 < ‐> −4000 bp upstream the TSS.

Intriguingly, although, up to 90% of the variation in adult height may be explained by genetic factors (Silventoinen et al. 2003; Weedon and Frayling 2008), stature‐associated polymorphisms have been found to only explain between 2% and 3.7% of height variation (Gudbjartsson et al. 2008; Lettre et al. 2008; Weedon and Frayling 2008; Weedon et al. 2008). More recent analyses have increased the combined predictive power of the identified traits (Yang et al. 2010). Nevertheless, a large fraction of heritable height‐associated factors has escaped detection by conventional GWAS (Gudbjartsson et al. 2008; Lettre et al. 2008; Weedon and Frayling 2008; Weedon et al. 2008; Yang et al. 2010), consistent with difficulties of previous association studies in finding variants robustly associated with height (Lettre et al. 2007; Weedon et al. 2007; Sanna et al. 2008; Mackay et al. 2009).

Notably, rather large cohorts were analyzed in the human height‐SNP association studies (Gudbjartsson et al. 2008; Lettre et al. 2008; Weedon et al. 2008; Cho et al. 2009), which were designed to provide adequate power of detection (approximately 500,000 SNP spaced across the genome (Barrett and Cardon 2006)). Specific cohorts reached an up to 98% power to detect variants associated with ≥0.5% of the height variation (Lettre et al. 2008). The significant overlaps in findings obtained by independent groups (23/87, i.e., 26.4% of the genes, were identified by more than one study) (Table 1, Table S1), supported the analytical power of the studies performed. In other words, a significant coverage of height‐associated variation appeared to have been reached, and a significant fraction of the most strongly height‐associated loci was actually identified. Nevertheless, a large fraction of heritable height appeared unaccounted for, indicating that a corresponding large fraction of information might have been systematically missed by SNP‐based GWAS. A candidate for such non‐DNA sequence‐linked information is epigenetic heredity. Strategies designed to detect sequence polymorphisms would, indeed, systematically miss genetic information that is not associated with DNA sequence changes.

Functionally‐relevant DNA methylation patterns were thus candidates to be associated with adult stature subgroups in addition to DNA sequence variants. Functionally‐relevant DNA methylation patterns may affect selective mechanisms, thus behaving as true hereditary traits. Consistent with this, a metastable epigenetic heredity of the DWARF1 locus was shown to affect plant size (Miura et al. 2009), and this phenotype was inherited through mitosis and meiosis. Notably, environmental conditions, nutrition in particular, have been shown to affect height (Silventoinen et al. 2000). DNA methylation patterns can keep record of the nutritional status (El‐Osta et al. 2008; Guerrero‐Bosagna et al. 2008) and affect, in turn, morphometric parameters (Guerrero‐Bosagna et al. 2008). Modifications of DNA methylation patterns in growth‐related genes can be inherited trans‐generationally (Guerrero‐Bosagna et al. 2008; Hollingsworth et al. 2008; Nadeau 2009; Roth et al. 2009; Braunschweig et al. 2012), through incomplete erasure of epigenetic patterning in the germline. An example of trans‐generational epigenetic heredity of complex traits is that of longevity in *Caenorhabditis elegans* (Greer et al. 2011). An accumulation of epigenetic changes through generations would then provide a valuable, reversible mechanism of adaptation to progressively changing environments (Nadeau 2009; Roth et al. 2009; Verginelli et al. 2009).

These findings led us to assess the functional relevance of trait‐associated DNA methylation patterns. Our results indicate that inheritance of CpG island methylation patterns may indeed be involved in the control of body development. They also suggest that environmental influence on height may be mediated by modulation of epigenetic heredity. This may help to account for progressive height changes over multiple generations, through trans‐generational heredity of progressive DNA methylation patterns.

## Material and Methods

### CpG island ‐ DNA methylation analysis

Most genes regulated by DNA methylation contain one or more CpG islands, most frequently in their promoter region (Lander et al. 2001; Saxonov et al. 2006; Esteller 2008; Illingworth and Bird 2009; Jin et al. 2012). The presence of CpG islands in the 87 genes most strongly associated with the variation in human height (Gudbjartsson et al. 2008; Lettre et al. 2008; Weedon et al. 2008; Cho et al. 2009) was investigated using capabilities of the USC CpG island searcher (cpgislands.usc.edu/) (Takai and Jones 2002). The human genome sequence assembly 36.3 was used (www.ncbi.nlm.nih.gov/sites/entrez). To exclude GC‐rich Alu repetitive elements, CpG island limits of >500 bp, ≥55% in G + C content and observed CpG/expected CpG >0.65 were imposed. Validation analyses were performed at the UCSC Genome Browser (genome.ucsc.edu/cgi‐bin/hgGateway) (Illingworth and Bird 2009).

Relevance for disease pathological development was investigated using OMIM (Online Mendelian Inheritance in Man) resources (omim.org).

### Gene imprinting

Genomic imprinting is the mechanism by which monoallelic expression is achieved in a parent‐specific fashion. Several human genes are known to be imprinted (Nazor et al. 2012). These comprise epigenetic changes, when the imprints are established in the germline, or somatic changes, when they arise during early embryonic development. Genomic imprinting defects are associated with developmental disorders, including Silver‐Russell, Beckwith‐Wiedemann, and Prader‐Willi syndromes. Genomic imprints are affected by environmental factors, and also associate with several human cancers. Gene imprints were then analyzed for paternal or maternal patterns and gene expression regulation (www.geneimprint.org).

### DNA hypermethylation modules

Association with DNA hypermethylation modules was evaluated as described (Easwaran et al. 2012). Briefly, at a genome‐wide level, hypermethylated genes are marked by Polycomb complex signatures. Most of these genes comprise developmental regulators, which contain DNA hypermethylation modules, within bivalent, that is divergently regulated, chromatin.

### DNA methylation dynamics

The status of DNA methylation of regulatory regions of height‐associated genes was assessed to provide information on DNA methylation dynamics (www.methdb.de/;202.97.205.78/diseasemeth/) (Lv et al. 2011).

### Regulation of height‐associated genes by DNA methylation

Mechanistic relevance of DNA methylation was assessed versus a regulatory role of promoter methylation on the expression levels of height‐associated genes.

### Signaling networks of height‐associated genes

**Figure 1.**
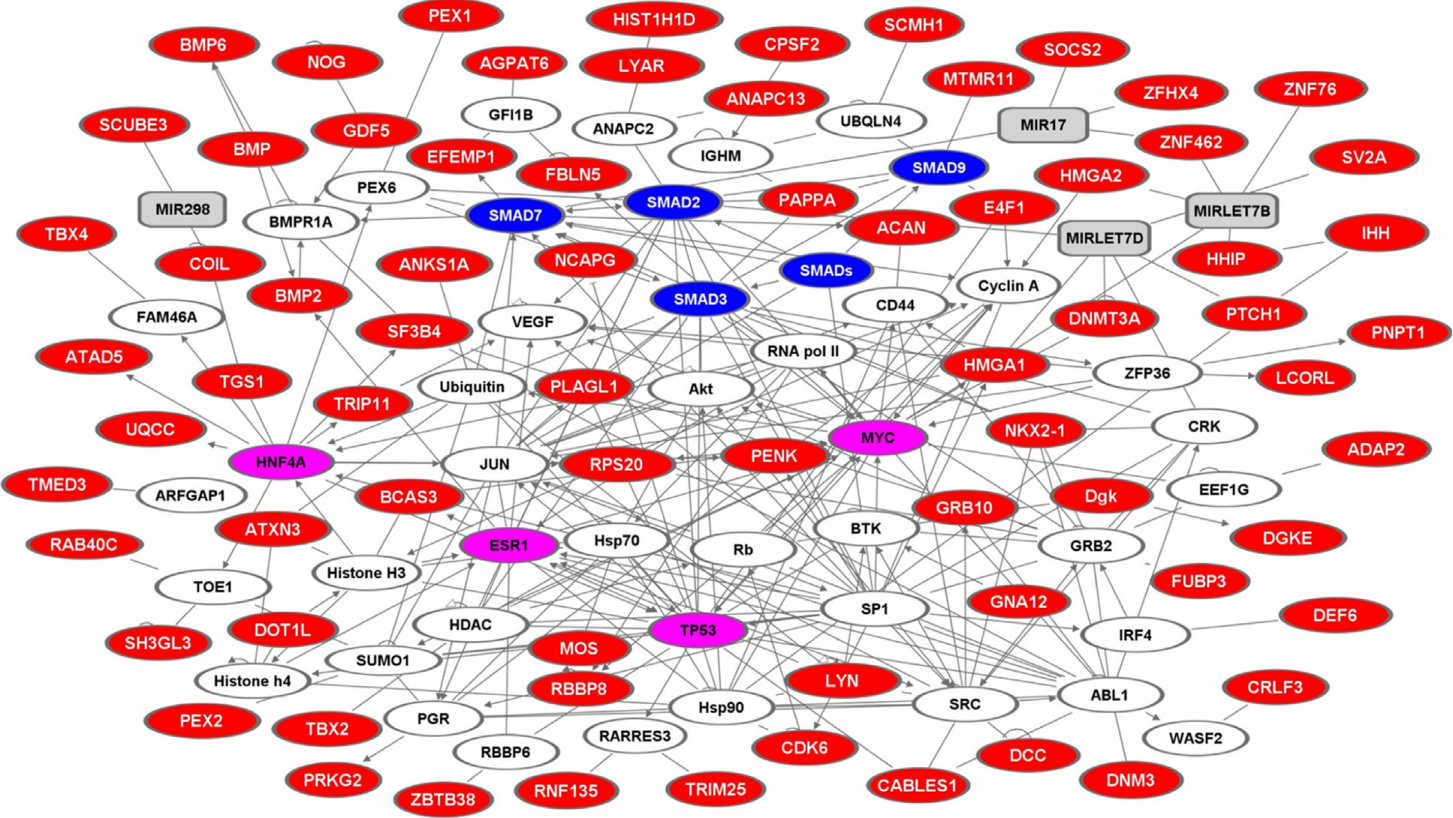
Pathway analysis. Graphical representation of the height‐associated proteins relationships retrieved through SNOW network analysis. Proteins are represented as nodes (hubs), the biological relationships between the nodes (edges) are represented as lines. Height‐associated proteins are in red; linker proteins are in white; miRNA are in gray. Major hubs are in magenta; SMAD isoforms are in blue.

To detect potential associations to cancer growth control pathways, network‐based analyses were designed:

SNOW (studying networks in the omics world; babelomics.bioinfo.cipf.es/) builds protein–protein contact networks and maps lists of genes or proteins over a reference interactome, where nodes are proteins and edges are interaction events. A SNOW interactome (*minimal connected networks*, MCN) of height‐associated genes was built using the HPRD (human protein reference database), IntAct, BIND (biomolecular interaction network database), DIP (database of interacting proteins) and MINT (molecular interaction database). Topological parameters included the distributions of node *connections degrees* (i.e., of the number of edges for each node); the *betweenness* (i.e., a measure of centrality of all nodes and of their distribution); the distribution of *clustering coefficients* (i.e., of the connectivity of the neighborhood of each node) and the *number of components* of the network (i.e., the different groups of nodes that are generated in a network analysis). MCNs were identified adopting the option of only one bridging protein between any pair of proteins analyzed. Statistical significance was computed by comparing the obtained networks with the entire reference interactome or with networks of random composition. All networks presented below have significantly higher betweenness and lower connections degrees as compared with irrelevant networks (*P* < 0.0001) (Fig. 1).

MetaCore^™^ network analysis (http://lsresearch.thomsonreuters.com/pages/solutions/1/metacore) builds on a curated database. MetaCore^™^ is based on a proprietary manually curated database of human protein‐protein, protein‐DNA and protein‐com pound interactions, metabolic and signaling pathways for human, mouse and rat, supported by proprietary ontologies and controlled vocabulary. Over 2000 multi step canonical pathway maps of human protein‐protein and protein‐DNA interactions are utilized as reference maps. The *analyze network TF* feature was exploited to identify transcriptionally regulated pathways. The *shortest pathway* algorithm was used to link the height‐associated genes with additional database objects along a directed path, enforcing the stringent option of one‐intermediate‐only.

Ingenuity pathways analysis (Ingenuity Systems, www.ingenuity.com) was utilized to map height‐associated genes onto the Ingenuity knowledge base (focus points). Networks of focus molecules were generated by maximizing specific connectivities. Networks were ranked by score (negative log *P*‐value by right‐tailed Fisher's exact test). Each score takes into account the number of molecules in the network, the final network size, as well as the dataset size and the total number of network‐forming molecules in the Ingenuity Knowledge Base.

Data meta‐analysis (Tables S1–S3) validated identity and relationships between network members (www.signaling-gateway.org/; www.wikipathways.org/index.php/Wiki-Pathways and www.genome.jp/kegg/pathway.html). Yes/no relevance for specific signaling pathways (SMAD; c‐Myc; p53; ER*α*) or processes (development, cell growth; apoptosis) was verified by wet‐lab approaches (www.ncbi.nlm.nih.gov/sites/entrez; and manuscript in prep.).

### Statistical analysis

Statistical significance of differences between groups was assessed by Fisher exact test (SISA, www.quantitativeskills.com/sisa/ and GraphPad Prism 6.02).

## Results

### DNA methylation of height‐associated genes

Most genes regulated by DNA methylation contain one or more CpG islands, most frequently in their promoter region (Lander et al. 2001; Saxonov et al. 2006; Esteller 2008; Illingworth and Bird 2009). The presence of CpG islands in the genes most strongly associated with the variation in human height (87 loci) (Gudbjartsson et al. 2008; Lettre et al. 2008; Weedon et al. 2008; Cho et al. 2009) was investigated (Takai and Jones 2002) (Table 1).

Remarkably, 72 of 87 height‐associated genes (82.8%) were found to contain at least one CpG island in the 2,000 bp upstream of the transcription start site (TSS) (99 CpG islands overall) (Table 1). Notably, in all CpG islands‐associated height genes, CpG islands overlapped with the TSS, supporting an actual regulatory role in gene transcription. As gene expression regulatory regions can significantly extend upstream of the TSS (Koudritsky and Domany 2008), we extended our analysis to the 2,000–4,000 bp upstream of the TSS (Table 1), and identified CpG islands in three additional genes (11 islands overall). More extensive investigation to 10,000 bp 5' from the TSS identified four additional genes as containing a CpG island (only 15 additional islands overall), suggesting adequate coverage of our search (Table 1 and Table S1). These observed frequencies (90.8% for 10 kb analysis) were strikingly higher than those measured across the whole genome (Jiang et al. 2007; Zhu et al. 2008). Housekeeping, that is universally expressed, genes contain CpG islands at higher‐than‐average frequency [78.7% (Larsen et al. 1992; Zhu et al. 2008)]. Thus, we repeated our analysis after removal of housekeeping height genes [five cases (Jiang et al. 2007; Zhu et al. 2008)] (Table 2). Nonhousekeeping height genes were confirmed to host CpG islands at a much higher‐than‐expected frequency [87.8% vs. 45% for nonhousekeeping genes (Jiang et al. 2007)] (P = 1.10e^−11^; Fisher exact test). It should be noted that most tissue‐specific/nonhousekeeping genes possess neither CpG‐islands nor TATA‐boxes in their core promoters, and only 19.2% of tissue‐specific genes have a TATA‐less CpG‐island associated core promoter (Zhu et al. 2008), further supporting the specificity of the association of height genes to CpG islands.

**Table 2.** Functional relevance of DNA methylation of human height‐associated genes.

Gene symbol	Gene name	DNA methylation	Notes^1^
*ACAN*	Aggrecan		
*ADAMTSL3*	ADAMTS like 3	Dunn et al. (2004)	*
*ADAMTS17*	ADAM metallopeptidase with thrombospondin type 1 motif 17	Dunn et al. (2004)	*
*AGPAT6*	1‐acyglycerol‐3‐phosphate O‐acyltransferase 6		
*ANAPC13*	Anaphase promoting complex subunit 13		
*ANKS1*	Ankyrin repeat and sterile alpha motif domain containing 1		
*ATAD5*	ATPase family AAA domain containing 5		
*ATXN3*	Ataxin 3		
*BCAS3*	Breast carcinoma amplified sequence 3		
*BMP2*	Bone morphogenic protein 2	Wen et al. (2006)	*
*BMP6*	Bone morphogenic protein 6	Taniguchi et al. (2008)	*
*CABLES1*	cdk5 and Abl enzyme substrate 1	Sakamoto et al. (2008)	*●
*CDK6*	Cyclin‐dependent kinase 6		
*CENTA2*	Centaurin alpha 2		
*CHCHD7*	Coiled‐coil‐helix‐coiled‐coil‐helix domain containing 7		
*COIL*	Coilin		
*CPSF2*	Cleavage and polyadenylation specific factor 2		**●**
*CRLF3*	Cytokine receptor like factor 3		
*DCC*	Deleted in colon carcinoma	Park et al. (2008)	*
*DEF6*	Differentially expressed in FDCP6 homolog		
*DGKE*	Diacylglycerol kinase epsilon 64 kDa		
*DLEU7*	Deleted in lymphocytic leukemia	Hammarsund et al. (2004)	*
*DNM3*	Dynamin 3		
*DNMT3A*	DNA methyl transferase 3 alpha	Esteller (2008)	‡
*DOT1L*	DOT1‐like,histone H3 methyltransferase	Jones et al. (2008)	‡
*DYM*	Dymeclin		
*EFEMP1*	EGF containing fibulin like extracellular matrix protein 1	Sadr‐Nabavi et al. (2009)	*
*E4F1*	E4F transcription factor 1		
*FBLN5*	Fibulin5		
*FUBP3*	Far upstream element (FUSE)‐binding protein 3		
*GATAD1*	GATA zinc finger domain containing 1		
*GLT25D2*	Glycosyltransferase 25 domain containing 2		
*GNA12*	Guanine nucleotide‐binding protein (G protein) alpha 12		**●**
*GPR126*	G protein coupled receptor 126		
*GRB10*	Growth factor receptor bound protein 10	Li et al. (2008)	*
*HHIP*	Hedgehog interacting protein	Tada et al. (2008)	*
*HIST1H1D*	Histone H1D	Albig et al. (1993)	‡
*HMGA1*	High mobility group AT‐hook 1	Sgarra et al. (2003)	‡
*HMGA2*	High mobility group AT‐hook 2	Reeves and Beckerbauer (2001)	‡
*IHH*	Indian hedgehog		
*LBH*	Limb bud and heart development homolog		
*LCORL*	Ligand‐dependent nuclear receptor corepressor‐like protein		
*LIN28B*	Lin 28 homolog B		
*LYAR*	Ly1 antibody reactive homolog		
*LYN*	v‐yes‐1 Yamaguchi sarcoma viral related oncogene homolog		
*MOS*	v‐mos Moloney murine sarcoma viral oncogene homolog	Scholz et al. (2005)	*
*MTMR11*	Myotubularin‐related protein		
*NACA2*	Nascent polypeptide‐associated complex alpha subunit 2		
*NCAPG*	Non‐SMC condensin I complex subunit G		
*NOG*	Noggin		
*NKX2‐1/TTF1*	NK2 homeobox/thyroid transcription factor 1	Kondo et al. (2009)	*
*PAPPA*	Pregnancy‐associated plasma protein A		
*PENK*	Proenkephalin	Comb and Goodman (1990)	*
*PEX1*	Peroxisome biogenesis factor 1		
*PLAGL1*	Pleomorphic adenoma gene 1	Arima and Wake (2006)	*
*PNPT1*	Polyribonucleotide nucleotidyltransferase		
*PRKG2*	Protein kinase, cGMP‐dependent, type II		
*PTCH1*	Patched homolog 1 (hedgehog signaling)	Wolf et al. (2007)	*
*PXMP3*	Peroxisomal membrane protein 3 35 kDa		**●**
*RAB40C*	Member RAS oncogene family		
*RBBP8*	Retinoblastoma‐binding protein 8	Li et al. (2014)	*
*RDHE2*	Epidermal dehydrogenase 2		
*RNF135*	Ring finger protein 135		
*RPS20*	Ribosomal protein S20		
*SCMH1*	Sex comb on midleg homolog 1		**●**
*SCUBE3*	Signal peptide CUB‐domain EGF‐like 3		
*SF3B4*	Splicing factor 3b subunit 4 49 kDa		
*SH3GL3*	SH3‐domain GRB2‐like 3		
*SOCS2*	Suppressor of cytokine signaling 2	Sutherland et al. (2004)	*
*SPAG1*	Sperm antigen 17		
*SV2A*	Synaptic vescicle glycoprotein 2A		
*TBX2*	T‐box 2		
*TBX4*	T‐box 4		
*TCP11*	t‐complex protein 11		
*TGS1*	Trimethylguanosine synthase homolog		
*TMED3*	Transmembrane emp24 protein transport‐domain containing 3		
*TRIM25*	Tripartite motif containing 25		
*TRIP11*	Thyroid hormone receptor interactor 11		
*UQCC/GDF5*	Ubiquinol cytochrome C reductase chaperone/growth differentiation factor 5		
*WDR60*	WD repeat domain 60		
*ZBTB38*	Zinc finger and BTB domain containing 38	Filion et al. (2006)	‡
*ZFHX4*	Zinc finger homeobox 4		
*ZNF76*	Zinc finger protein 76		
*ZNF462*	Zinc finger protein 462		
*ZNF678*	Zinc finger protein 678		

^1^ *Genes regulated by DNA methylation; ^‡^Genes regulating DNA methylation; **^●^**Housekeeping genes.

As an internal control, searches for CpG islands were conducted in the transcribed region of height‐associated genes. The regulatory force of CpG islands in transcribed regions is lower than that of islands upstream the TSS (Nguyen et al. 2001; Weber et al. 2007). Consistently, only 35 height‐associated genes (40.23% of the total) were found to contain CpG islands in transcribed segments (Table S1), which did not exceed the expected frequency. This supported the relevance of a quasi‐universal presence of CpG islands in the promoter of height‐associated genes.

Independent searches for CpG islands were conducted with the UCSC Genome Browser, using broader/less stringent settings [length of ≥200 bp and for G+C content of ≥50% (Illingworth and Bird 2009)] (Table S2). Largely concordant results were obtained with the USC CpG Island Searcher and the UCSC Genome Browser algorithms. The highest concordance was found in the promoter region (84% of the cases), consistent with the actual identification of *bona fide* transcriptionally active CpG islands.

### Gene imprinting

Gene imprinting was analyzed for paternal or maternal patterns and regulation of gene expression (www.geneimprint.org). Listed in Table S3 are the height‐associated genes/gene clusters that were found associated with imprinting (Nazor et al. 2012). Twenty five height‐associated genes were found to be imprinted. Additionally, imprinting of *GNAS* was shown to lead to either gynogenetic or androgenetic patterns. Growth factor receptor bound protein 10 (*GRB10*) expression was shown an imprinted gene (Li et al. 2008). *PLAGL1* lies within a DNA methylation imprinting center on human chromosome region 6q24. Imprinting of *PLAGL1* was also shown by expression and phenotypic profiling of parthenogenetic fetuses (Bischoff et al. 2009). Hypomethylation patterns were demonstrated for both *PLAGL1* and *GRB10*. Using Affymetrix GeneChip microarrays (Carletti et al. 2006) and/or semiquantitative PCR, organs expression patterns were profiled, and *PLAGL1* was found expressed in a tissue‐specific manner; imprinting was confirmed by pyrosequencing. A paternally imprinted regulatory role was also shown, as paternal noncoding RNAs at this locus can interact with regulators of active chromatin (Iglesias‐Platas et al. 2012).

### DNA hypermethylation modules

The functional relevance of DNA methylation of height‐associated genes was first assessed by association with DNA hypermethylation modules (Easwaran et al. 2012) (Table S3), as marked by Polycomb‐complex signatures within bivalent chromatin. Most of Polycomb‐signed genes comprise developmental regulators, and were shown to contain DNA hypermethylation modules (Easwaran et al. 2012). As compared to control H3K4Me3 genes, which are hypermethylated in 1.5% of cases (Easwaran et al. 2012), DNA hypermethylation modules were detected in 42/87 height‐associated genes (P = 8.02e^−17^; Fisher exact test, two tailed).

### DNA methylation dynamics

The extent of DNA methylation of regulatory regions of height‐associated genes was experimentally assessed as described by Lv et al. (2011) (www.methdb.de/; 202.97.205.78/diseasemeth/) (Table S3). All genes where DNA methylation plays a regulatory role are expected to undergo shifts in methylation states. We challenged this prediction in our gene‐set. Only five genes (*ACAN, ANKS1, FBP2, NACA2, ZBTB38*) were found to have no evidence of DNA methylation. The remaining genes (94.3%) were shown to undergo broad changes of DNA methylation levels across experimental conditions (Table S3), consistent with shifting between distinct, DNA methylation‐associated regulatory states.

### Regulation of height‐associated genes by DNA methylation

A mechanistic relevance of these findings was investigated.

All assessed genes were shown to possess a CpG island in the promoter region that overlaps the TSS (Tables S1, S2), consistent with functional relevance on gene transcription (Esteller 2008).

Loss of ADAMTS proteases in nonsmall‐cell lung carcinomas is caused by hypermethylation in the *ADAMTS* genes promoters (Dunn et al. 2004).

BMPs are important regulators of cell growth, differentiation, and apoptosis. CpG island methylation in the *BMP2* promoter causes loss of BMP‐2 protein expression in transformed cells (Wen et al. 2006). Shut‐down of the *BMP6* gene by promoter methylation was observed in malignant lymphomas (Taniguchi et al. 2008).

Cdk5 and Abl enzyme substrate 1 (*CABLES1*) is a cyclin‐dependent kinase binding protein. Loss of nuclear CABLES1 expression is due to epigenetic modifications of the *CABLES1* locus. Full ablation is caused by LOH of the transcriptionally‐competent allele (Sakamoto et al. 2008).

The expression of the deleted in colorectal cancer (*DCC*) gene is frequently lost in intestinal cancers. In up to three quarters of the cases the loss of expression of *DCC* is due to DNA methylation (Park et al. 2008).

Loss of the deleted in lymphocytic leukemia 7 (*DLEU7*) gene is frequently observed in chronic lymphocytic leukemia, due to hypermethylation of the *DLEU7* promoter (Hammarsund et al. 2004).

Decrease or loss of EGF‐containing fibulin‐like extracellular matrix protein 1 (EFEMP1) has been shown in human breast cancer. *EFEMP1* promoter methylation is a major cause of down‐regulation (Sadr‐Nabavi et al. 2009).

Hedgehog (Hh) signaling activation is frequently mediated by the epigenetic repression of the Hh‐interacting protein (*HHIP*) gene, a negative regulator of Hh signaling. Consistently, *HHIP* transcription is rescued by demethylation (Tada et al. 2008).

Hypermethylation of *MOS* is associated with the development of acute lymphoblastic leukemia (Scholz et al. 2005).

CpG methylation inhibits proenkephalin (*PENK*) gene expression by interfering with the binding of the stimulatory transcription factor AP‐2 (Comb and Goodman 1990).

Differential methylation of the zinc finger protein gene pleomorphic adenoma gene 1 (*PLAGL1*) was found in diabetes and cancer and was shown to modulate the expression levels of this gene (Arima and Wake 2006).

Patched homolog 1 (PTCH1) is the Hh receptor. Methylation of the *PTCH1* promoter inhibits *PTCH1* expression in breast cancers (Wolf et al. 2007).

The suppressor of cytokine signaling (*SOCS*) 2 CpG islands are hypermethylated in ovarian cancers. Aberrant methylation of this gene correlates with transcriptional silencing also in hepatocellular carcinomas (Martinez‐Chantar et al. 2008).

DNA methylation suppresses the expression of thyroid transcription factor‐1 (TTF‐1) in 60% of undifferentiated thyroid carcinomas (Kondo et al. 2009).

c‐Myc is a widely expressed TF, that can bind 10–15% of all genes (Orian et al. 2003). c‐Myc regulates at least seven height‐associated genes (*CDK6, COIL, HMGA1, LIN28B, RBBP8, RPS20, TRIM25/EFP*), and its binding to genomic loci is dependent on chromatin structure and CpG methylation.

### Epigenetic defects and hereditary growth anomalies

Distinct epigenetic defects have been linked to separate hereditary growth anomalies, providing evidence for a broad regulatory role of DNA methylation on body growth. The Beckwith‐Wiedemann syndrome (130650) is caused by deregulation of imprinted genes within the 11p15 chromosomal region, i.e., KIP2, H19 and LIT1, whether alone or as interacting regulatory units (Niemitz et al. 2004). Hypermethylation at the 11p15 telomeric imprinting control region (ICR1), are observed in about 5 to 10% of affected patients (see below for opposite epigenetic changes in Silver‐Russel patients). Both H19 and LIT1, which encode untranslated RNAs, and IGF2 are either maternally imprinted genes with growth enhancing activity or paternally imprinted genes with growth suppressing activity. Key features of the syndrome are exomphalos, macroglossia, and gigantism in the neonate, supporting a direct involvement of epigenetic regulatory mechanisms in body size and body development. Consistently, alterations include visceromegaly, adrenocortical cytomegaly and overgrowth of the external genitalia in both males and females. Affected children reach an average height of 2.5 SD above the mean at or after puberty, and their growth velocity is above the ninetieth percentile until 4–6 years of age.

Prader–Willi syndrome (176270) is a complex childhood disorder that affects the nervous and hormonal systems and leads to excessive weight gain. It arises from the loss of activity of several imprinted genes on chromosome 15, that are usually expressed from the paternally inherited copy. Affected children suckle poorly for the first months of their lives; their voracious appetite and consequent obesity develop from weaning onwards.

Angelman's syndrome (AS) (105830) arises from opposite imprinting defects to those described in Prader–Willi syndrome. The Angelman syndrome is characterized by mental retardation, movement disorders, altered behavior, and speech abnormalities. Most cases are caused by absence of a maternal contribution to the imprinted region on chromosome 15q11‐q13, whereas the Prader‐Willi syndrome results from deletion of the same region in the paternal chromosome. Other patients with an Angelman syndrome carry mutations in the gene encoding methyl‐CpG‐binding protein‐2 (MECP2).

MECP2 (300005) is the gene mutated in the Rett syndrome, a neurological (regression of acquired skills, loss of speech, stereotypical movements, seizures, and mental retardation) and developmental disorder that occurs in females. Notably, affected girls are characterized by microcephaly and arrested development between 6 and 18 months of age.

Imprinting defects are associated with other developmental disorders, for example Silver‐Russell dwarfism. Up to 60% of cases of Silver‐Russell syndrome are caused by hypomethylation at the ICR1 on chromosome 11p15, involving the H19 (103280) and IGF2 (147470) genes (Penaherrera et al. 2012). Affected patients demonstrate severe intrauterine growth retardation, poor postnatal growth, craniofacial alterations, and a variety of minor malformations.

### Epigenetic regulation of body size

The findings on Beckwith‐Wiedemann syndrome, Prader–Willi syndrome, Angelman's syndrome, Rett syndrome and Silver‐Russell syndrome suggest an epigenetic regulation of body size. Consistently, several other imprinting disorders have been shown to affect placental and fetal size (Constancia et al. 2004). The connection between imprinting and placentation is supported by the finding that erasing all genomic imprints results in the outgrowth of extra‐embryonic tissues and placenta in animal models. Correspondingly, imprinting defects in the female human germline cause the appearance of hydatidiform moles, that is uncontrolled growth of placental cells.

### Height‐associated regulators of epigenetic heredity

Height‐associated genes include several key epigenetic regulators of gene expression (Esteller 2008). The activity of transcription factors is modulated by associations with co‐repressors, including histone deacetylase 7. BMP2 regulates the transcriptional activity of HDAC7 by inducing its export from the nucleus. The zinc finger and BTB domain containing (ZBTB38) is a methyl‐DNA‐binding transcriptional repressor gene that binds single methylated CpGs. Chromatin immunoprecipitation indicates that ZBTB38 recognizes the methylated alleles of the H19 and insulin‐like growth factor II (IGF‐2) genes and represses their transcription (Filion et al. 2006). Methylation‐linked inactivation of IGF‐2/H19 can be inherited in a parental‐specific manner (Riccio et al. 2009).

The DOT1‐like and NSD1 histone methyltransferases, the two high‐mobility group A (HMGA) genes, HMGA1 and HMGA2, and the histone clusters 1 and 2 are all involved in the assembly of chromatin structure. Notably, haploinsufficiency of the histone methyltransferase NSD1 causes the Sotos syndrome, that is characterized by very high stature (Kurotaki et al. 2002) and the gene can be inactivated epigenetically in tumors (Berdasco et al. 2009). PRMT5 contributes to coupling DNA methylation and chromatin organization *via* the methylation of histone H4R3, which then recruits DNMT3A. DNMT3A mediates additional regulatory loops in chromatin organization, as the HMGA1 gene itself is regulated by DNA methylation.

Heterozygous 17q11 microdeletions that encompass NF1 and RNF135 cause a subset of neurofibromatosis type 1 (Douglas et al. 2007). Notably, individuals with microdeletions are typically taller than individuals with NF1 mutations, consistently with the removal of a gene that negatively regulates human growth (RNF135 haploinsufficiency) (Douglas et al. 2007).

### Prenatal environmental factors and persistence of DNA methylation changes in the adult

Epigenetic patterns in germ cells can persist into adulthood, and many imprinted genes continue to be expressed from a single parental copy. Environmental factors during pregnancy affect epigenetic marks, and in utero conditions have consequences on adult health and disease (Gluckman et al. 2008). This has been proven due to DNA methylation differences after exposure to prenatal famine (Tobi et al. 2009). Animal studies and human data on the imprinted *IGF2* locus indicated a link between prenatal nutritional and DNA methylation. Additionally, methylation of the *INSIGF*,* IL10*,* LEP*,* ABCA1, GNASAS* and *MEG3* genes was persistently modified by exposure to prenatal famine (Tobi et al. 2009).

In animal models manipulation of the maternal diet during pregnancy leads to a persistent shift in average DNA methylation levels of specific genes in offspring resulting in permanent changes in coat color or tail shape. Moreover, widespread dynamics of DNA methylation were shown in response to biotic stress (Dowen et al. 2012). Methyl donor supplementation prevents transgenerational amplification of obesity (Waterland et al. 2008) and maternal methyl supplements were shown to increase DNA methylation in offspring (Waterland et al. 2006).

### Epigenetic control of height‐associated genes networks

Data‐bank web network meta‐analyses allowed us to connect height‐associated genes in a function‐driven web. These identified p53, c‐Myc, estrogen receptor alpha (ER*α*), HNF4A (Guerra et al. 2012; Trerotola et al. 2013) and SMADs as major hubs (Fig. 1). The Hh pathway and regulatory clusters for programmed cell death/apoptosis were also identified as key control pathways, that are shared between human height and cancer.

*MYC:* Metacore analysis revealed that the c‐Myc regulates at least seven height‐associated genes (*CDK6, COIL, HMGA1, LIN28B, RBBP8, RPS20 and TRIM25/EFP*). A SNOW analysis showed that c‐Myc is a major hub of the height‐associated protein network (19 connections, betweenness 0.366). Thirty‐seven height‐associated proteins were shown to be associated in the network. The binding of c‐Myc to genomic loci is highly dependent on chromatin structure and DNA methylation (Guccione et al. 2006). c‐Myc modulates gene expression also by increasing methylation of the 5′ mRNA guanine or ‘cap’, which regulates the translation of individual mRNAs. Methylation of the cap is required for eukaryotic translation initiation factor‐4E binding and recruitment onto ribosomes for translation (Cole and Cowling 2008). c‐Myc regulates the cell cycle, and plays a major role in cell growth during interphase, by regulating genes required for the production of energy and metabolites. The c‐Myc network widely interacts with those driven by other major hubs. c‐Myc is repressed by transforming growth factor *β* (TGF‐*β*) through the binding of SMAD3 to the *MYC* promoter (Frederick et al. 2004). p53 represses c‐Myc through the induction of the tumor suppressor miR‐145 (Sachdeva et al. 2009). c‐Myc amply interacts also with the ER network: almost all of the acutely estrogen‐regulated genes with roles in cell growth are c‐Myc targets. Notably, estrogen‐mediated activation of rRNA and protein synthesis depends on c‐Myc (Musgrove et al. 2008). Equally c‐Myc dependent is the estrogen‐induced suppression of apoptosis caused by growth factor deprivation (Rodrik et al. 2005).

*TP53*: A major hub of height‐associated genes is *TP53*. Remarkably, at least 47 of the 87 height‐associated genes were identified as members of p53 signaling networks (Table 1), including 36 *via* direct protein–protein contacts. p53 was the most important hub (25 connections, betweenness 0.446). Metacore analysis similarly identified p53 as a hub of the height‐associated genes network. Input of p53 in the *shortest pathway* analysis indicated that p53 has a central role with a total of 125 edges pointing either in (37) or out (88) from it. Among its functions, p53 regulates the expression of target genes that modulate chromatin structure and function (Cimoli et al. 2004; Vousden and Lane 2007), cell growth, aging and apoptosis (Ambrogi et al. 2006; Biganzoli et al. 2011). p53 interacts with components of multiple different histone remodeling complexes, including CBP/EP300 (CBP/p300), GCN5, PCAF, and SETD7 modifying histones at the promoters [(Kaneshiro et al. 2007) and reference therein]. We have previously shown that p53 also controls DNA methylation levels, and that this affects genome stability (Alberti et al. 1994; Nasr et al. 2003). p53 is involved in bone remodeling, wound healing and neural tube development (Vousden and Lane 2007). It also performs an anti‐teratogenic function, reducing the rate of birth defects, through the induction of apoptosis of aberrant cells (Vousden and Lane 2007). Defects of induction of apoptosis play a role also at later stages of development [(Rossi et al. 2008) and references therein]. Female *p53*‐null mice can present neural tube‐closure defects, due to a failure to induce apoptosis of progenitor cells. This is caused by abnormal regulation of mitochondrial death pathways and results in overproduction of neural tissue. p53 has a role in the regulation of both glycolysis and oxidative phosphorylation. Low levels of p53 determine a switch from oxidative phosphorylation to glycolysis. p53 enhances oxidative phosphorylation by inducing the expression of the cytochrome c oxidase 2 subunit 1 and of the ribonucleotide reductase subunit p52R2, which are involved in the maintenance of mitochondrial DNA. p53 promotes cell survival under conditions of low or basal stress. The reduction in nutrient or energy levels causes a failure to stimulate the AKT–mTOR pathway and induces the activation of AMPK both of which lead to the induction of p53.

*ESR1*: ER*α* is a transcription factor which binds to estrogen response elements upstream of the target genes. METACORE analysis shows that ER*α* regulates at least eight height‐associated genes (*BCAS3, BMP2, BMP6, DCC, GLT25D2, PENK, RBBP8, TRIM25/EFP*). ER*α* is a major hub (16 connections, betweenness 0,376). Estrogen is candidate to be the principal hormone stimulating the pubertal growth spurt in boys as well as girls (Juul 2001). This action is mediated by both ER*α* and ER*β*. Polymorphisms in the *ER* gene may influence adult height in healthy male and female subjects (Schuit et al. 2004; Dahlgren et al. 2008). Consistently, men with a disruptive mutation in the ER gene have no pubertal growth spurt and continue to grow into adulthood. ER*α* blockade diminishes the secretion of endogenous growth hormone, the key hormone regulator of linear growth in childhood (Juul 2001). This action is mediated by SOCS‐2 (Leung et al. 2003). The ER*α* network widely interconnects with the p53, Hh and BMP/TGF‐*β* pathways. p53 regulates ER expression through transcriptional control of the *ER* promoter (Shirley et al. 2009). Of interest, DNA methyltransferase expression in the human endometrium is down‐regulated by progesterone and estrogen (Yamagata et al. 2009). Estrogen receptor‐negative human breast cancer cells inactivate the promoter of the *ESR1* gene by methylation of its CpG island (Ottaviano et al. 1994). Correspondingly, DNA methylation‐mediated gene silencing is the mechanism of ER*α* inactivation in prostatic epithelial cells (Lau et al. 2000).

The SNOW‐connection analysis revealed that 33 of the height‐associated proteins take part in a unique network of protein–protein contacts (Fig. 1). Relevant hubs in this network are *COIL* (seven connections, betweenness 0.247) and *LYN* (seven connections, betweenness 0.146), followed by *GRB10* (six connections, betweenness 0.140) and *SF3B4* (six connections, betweenness 0.102). Coilin is a key constituent of Cajal bodies, which are responsible for the biogenesis of spliceosomal small nuclear ribonucleoprotein (snRNP) (Whittom et al. 2008). Cajal bodies are the initial nuclear sites for the assembly of macromolecular complexes involved in RNA processing and RNA transcription (Bogolyubov et al. 2009). Lyn is a Src tyrosine‐kinase family member. Signaling molecules phosphorylated by Lyn are the PI3‐kinase, STAT5 and MAP kinase. Grb10 is an adaptor protein that binds to phosphorylated tyrosines, for example, in the insulin receptor (IR) in response to insulin stimulation. Grb10 knockout mice show embryo and placenta overgrowth and a larger size at birth [(Wang et al. 2007) and references therein]. SF3b4 is a member of the spliceosome complex U2 snRNP and contributes to the recognition of the intron's branch point. SF3b4 also binds the BMPR‐IA serine/threonine kinase receptor and specifically inhibits BMP‐mediated SMAD1/5/8 pathway important for osteochondral cell differentiation.

## Discussion

Genomic loci linked to human height have been identified by genome‐wide SNP‐association analysis (Gudbjartsson et al. 2008; Lettre et al. 2008; Weedon et al. 2008). Corresponding GWAS in Korean (Cho et al. 2009) and Japanese (Okada et al. 2010) cohorts identified overlapping gene‐sets (Weedon and Frayling 2008), showing a conserved association with height in populations of both Caucasian and non‐Caucasian ancestry.

Intriguingly, although, albeit up to 90% of variation in adult height is explained by genetic factors (Silventoinen et al. 2003; Weedon and Frayling 2008), stature‐associated polymorphisms were found to only explain <10% of height variation (Gudbjartsson et al. 2008; Lettre et al. 2008; Weedon and Frayling 2008; Weedon et al. 2008; Eichler et al. 2010; Lango Allen et al. 2010). More recent analyses have increased this predictive power (Yang et al. 2010). However, a large fraction of heritable height‐associated factors still escapes detection by conventional GWAS (Gudbjartsson et al. 2008; Lettre et al. 2008; Weedon and Frayling 2008; Weedon et al. 2008; Eichler et al. 2010; Lango Allen et al. 2010; Yang et al. 2010; Lanktree et al. 2011), suggesting systematic loss of information by SNP‐based analyses. Candidate for such non‐DNA sequence‐linked information is epigenetic heredity.

Our findings indicate that most of the height‐associated genes contain CpG islands and their transcriptional activity is regulated by DNA methylation. Distinct epigenetic defects have been linked to hereditary growth anomalies, indicating a broad regulatory role of DNA methylation on body growth. Further support is now provided by the association of DNA methylation/regulatory transcription to genes involved in normal body growth (Gudbjartsson et al. 2008; Lettre et al. 2008; Weedon and Frayling 2008; Weedon et al. 2008; Eichler et al. 2010; Lango Allen et al. 2010; Yang et al. 2010; Lanktree et al. 2011). The DNA methylome thus appears to contain an as yet untapped fraction of heritable traits associated with human height, that may be exploited by genome‐wide analysis of DNA methylation patterns (Liu et al. 2013).

DNA methylation patterns are faithfully propagated through successive cell division cycles (Alberti and Herzenberg 1988; Esteller 2008). Some of these patterns can be inherited across generations (Kaminsky et al. 2009; Nadeau 2009; Roth et al. 2009; Braunschweig et al. 2012), thus becoming true heritable traits. DNA methylation can regulate gene expression, through the inhibition/activation of gene transcription of methylated/unmethylated genes, respectively (Alberti and Herzenberg 1988; Esteller 2008), and genome stability/recombination (Alberti et al. 1994; Nasr et al. 2003).

As DNA methylation patterns are affected by environmental stimuli (El‐Osta et al. 2008; Guerrero‐Bosagna et al. 2008), this mechanism allows for a dynamic and reversible modulation of the functional content of the genome (Esteller 2008), in the absence of DNA sequence variation. This may help explain as yet unaccounted for multi‐generational stature trends, for example, decrease in stature in agricultural populations compared to their paleolithic predecessors (Verginelli et al. 2009), and the recent increase in average height (Cole 2003).

## Acknowledgments

We thank R. Tripaldi, D. D' Ostilio and L. Apicella for support during the course of this work.

## Conflict of Interest

None declared.

## Supplementary Material

**Table S1**. CpG islands in human height-associated genes transcribed regions

**Table S2**. CpG islands in human height-associated genes - UCSC Genome Browser algorithm.

**Table S3**. Structural an functional features of DNA methylation in height-associated genes
